# Successive perioperative management of laparoscopic liver resection in the reverse Trendelenburg position for a patient with Fontan physiology: a case report

**DOI:** 10.1186/s40981-021-00456-6

**Published:** 2021-07-13

**Authors:** Kazutomo Saito, Hiroaki Toyama, Moeka Saito, Masanori Yamauchi

**Affiliations:** 1 Anesthesiology and Perioperative Medicine, Tohoku University Graduate School of Medicine, 2-1 Seiryomachi, Aoba-ku, Sendai, Miyagi 980-8575 Japan; 2 Department of Anesthesiology, Tohoku University Hospital, 1-1 Seiryomachi, Aoba-ku, Sendai, Miyagi 980-8575 Japan

**Keywords:** Laparoscopic liver resection, Reverse Trendelenburg position, Fontan-associated liver disease, Transpulmonary thermodilution

## Abstract

**Background:**

Laparoscopic surgery for a patient with Fontan physiology is challenging because pneumoperitoneum and positive pressure ventilation could decrease venous return and the accumulated partial pressure of arterial carbon dioxide (PaCO_2_) could increase pulmonary vascular resistance, which might lead to disruption of the hemodynamics.

**Case presentation:**

A 25-year-old man with Fontan physiology was scheduled to undergo laparoscopic liver resection for Fontan-associated liver disease (FALD) with noninvasive monitoring of cardiac output (CO) by transpulmonary thermodilution in addition to transesophageal echocardiography. The abdominal air pressure was maintained low, and we planned to switch to open abdominal surgery promptly if hemodynamic instability became apparent because of the accumulated PaCO_2_ or postural change. Consequently, the pneumoperitoneum had limited influence on circulatory dynamics, but central venous pressure significantly decreased with postural change to the reverse Trendelenburg position. Laparoscopic liver resection for FALD was performed successfully with no significant changes in CO and central venous saturation.

**Conclusions:**

With strict circulation management, laparoscopic surgery for a patient with Fontan physiology can be performed safely. Comprehensive hemodynamic assessment by noninvasive transpulmonary thermodilution can provide valuable information to determine the time for shift to open abdominal surgery.

## Background

Laparoscopic liver resection is usually performed in the reverse Trendelenburg position, which can induce a decrease in venous return from the lower extremity. The decreased preload can disrupt the hemodynamics of the Fontan circulation [1]. Because of CO_2_ insufflation and postural change, intraoperative management during laparoscopic liver resection after the Fontan procedure is not clearly described.

Meanwhile, open upper abdominal surgery is more invasive than laparoscopic surgery [2], and postoperative respiratory function of obese patients may worsen. Additionally, insufficient pain control and postoperative atelectasis, followed by hypoxia, can increase the pulmonary vascular resistance (PVR); therefore, minimally invasive surgery is preferable for obese patients with Fontan physiology.

In this report, we present the intraoperative management during laparoscopic liver resection for Fontan-associated liver disease (FALD) with noninvasive monitoring of cardiac output (CO) and transesophageal echocardiography.

## Case presentation

The patient was a 25-year-old obese man (height, 159.8 cm; body weight, 92.5 kg). At birth, he was diagnosed with double-outlet right ventricle, pulmonary atresia, and atrioventricular septal defect after cyanosis and heart murmur were evident. He underwent a right-modified Blalock-Taussig shunt at 34 days of age to increase the pulmonary blood flow. Cardiac catheterization indicated that the left ventricle was hypoplastic (left ventricular end-diastolic volume, 58% of normal), and pediatric cardiologists clarified the impossibility of biventricular repair. Therefore, he underwent the Glenn procedure at the age of 1 year and a fenestrated total cavopulmonary connection at the age of 3 years.

In this patient, the systemic ventricle was a morphologic right ventricle with a low ejection fraction, and the central venous pressure (CVP) was high at 16 mmHg; therefore, he was at a greater risk of hemodynamic instability.

At the age of 20 years, abdominal ultrasonography detected a large (>6 cm) tumor in segment 6 of the liver. Surgical treatment in Fontan-palliated patients is deemed unpredictable; therefore, he was treated non-surgically. However, at 25 years, protein induced by vitamin K antagonist-II level increased to 336 mAU/ml (cutoff value, 40 mAU/ml). Hepatocellular carcinoma was suspected; therefore, he was admitted to our hospital for surgical liver resection. On admission, the Child-Pugh class was determined as A. Blood hemoglobin was 17.3 mg/dl, platelet count was 21.3 × 10^4^/μl, and prothrombin time was 82.3%. The liver function was well preserved. Preoperative echocardiography revealed that the right-ventricular ejection fraction was 34% with a limited atrioventricular valvular regurgitation; CVP was 16 mmHg, saturation of peripheral oxygen (SpO_2_) was 94% on room air at rest, and PVR was low (1.0 WUm^2^).

It is difficult to determine whether laparoscopic surgery or open laparotomy procedure is more advantageous for a patient with Fontan physiology. Considerable preoperative discussions were conducted among the medical team members. In patients with Fontan physiology, adequate intravascular volume (preload) and low PVR should be maintained during the surgical procedure. During laparoscopic surgery, hypercarbia due to CO_2_ insufflation and high airway pressure caused by obesity could increase PVR. However, postoperative pain after open surgery could also increase PVR; therefore, we selected the less-invasive laparoscopic surgery for this Fontan-palliated patient. During pneumoperitoneum, this patient was scheduled to inhale nitric oxide, which dilates the pulmonary blood vessels selectively. Moreover, CO was measured continuously by transpulmonary thermodilution, such as a pulse contour cardiac output catheter (PiCCO; PULSION Medical Systems, Munich, Germany), in addition to transesophageal echography.

We planned to maintain low abdominal air pressure during pneumoperitoneum and to shift promptly to open abdominal surgery if hemodynamic instability became apparent because of the accumulated partial pressure of carbon dioxide (PaCO_2_) or postural change.

In the operating room, the patient’s electrocardiogram, SpO_2_, and noninvasive blood pressure (NIBP) were monitored. At baseline, NIBP was 132/88 mmHg, heart rate was 91/min, and SpO_2_ was 93% under room air conditions. After insertion of epidural catheter, general anesthesia was induced by propofol (90 mg), remifentanil (0.2 μg/kg/min), and rocuronium (100 mg). After orotracheal intubation, anesthesia was maintained by desflurane inhalation and fentanyl and remifentanil infusion. He received volume-controlled ventilation with fraction of inspired oxygen (F_I_O_2_) of 0.7, positive end-expiratory pressure of 6 cmH_2_O, peak inspiratory pressure of less than or equal to 22 cmH_2_O, tidal volume of 450 ml, and respiratory rate of 15–18 breaths/min. Minute ventilation was adjusted to maintain end-tidal CO_2_ (EtCO_2_) at 30–35 mmHg.

**Fig. 1:**
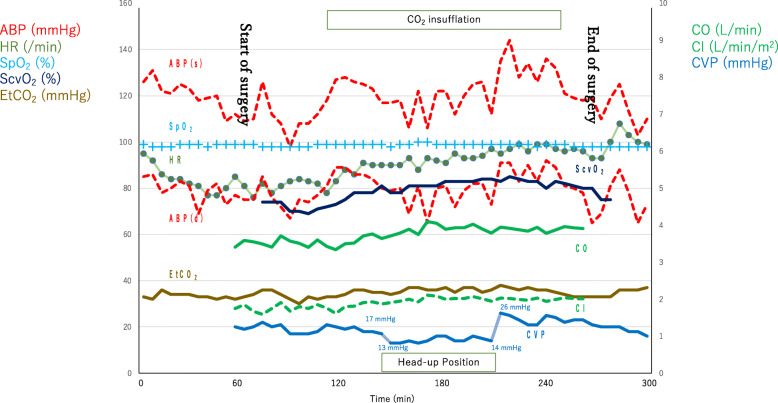
Trends of hemodynamic parameters during anesthesia. ABP, arterial blood pressure; HR, heart rate; SpO_2_, saturation of peripheral oxygen; ScvO_2_, central venous saturation; EtCO_2_, end-tidal CO_2_; CO, cardiac output; CI, cardiac index; CVP, central venous pressure

With ultrasound guidance, CO and stroke volume variant were monitored via the PiCCO catheter. Then, a central venous catheter (PreSep Catheter^TM^; Edwards Lifesciences, Irvine, CA, USA) was placed via the right internal jugular vein to monitor CVP and central venous saturation (ScvO_2_) continuously. The tip of the central venous catheter was located just cephalad to the connection between the superior vena cava and the right pulmonary artery. Figure 1 shows the trend of the vital signs, including CO, cardiac index (CI), CVP, ScvO_2_, and EtCO_2_ during anesthesia. Time 0 was defined as the start of general anesthesia. At baseline, F_I_O_2_ was 0.7, CI was 1.75 l/min/m^2^, CVP was 20 mmHg, and ScvO_2_ was 74%.

After initiating pneumoperitoneum, arterial blood pressure (ABP) increased slightly, but CO and ScvO_2_ were invariant. After postural change to the reverse Trendelenburg position, CVP decreased from 17 to 13 mmHg, but CO and ScvO_2_ remained unchanged. The insufflation pressure was maintained at 8–10 cmH_2_O. We administered an appropriate muscle relaxant for optimum surgical conditions. During pneumoperitoneum, the patient inhaled nitric oxide to decrease PVR. The Pringle maneuver during the liver resection did not affect CVP adversely. After withdrawal of the reverse Trendelenburg position, the CVP increased rapidly from 14 to 24 mmHg, but decreased subsequently to 20 mmHg. Nevertheless, CO and ScvO_2_ remained stable. CI and CO were stable at low levels, and laparoscopic partial hepatectomy was concluded successfully with hemodynamic stability. Transesophageal echography revealed no remarkable changes. Blood loss and urine output were 295 ml and 158 ml, respectively, which were replaced with 2200 ml of crystalloid.

After reversal of neuromuscular blockade, the patient was extubated and admitted to the intensive care unit. Postoperative analgesia using levobupivacaine and fentanyl was successfully administered via an epidural catheter. He remained hemodynamically stable and was shifted to the general ward on the 1st postoperative day. He was discharged on the 8th postoperative day with no postoperative complications. The resected liver mass was pathologically diagnosed as focal nodular hyperplasia with no malignancy.

## Discussion

With the improvement in surgical techniques and perioperative management, adult patients with postoperative congenital heart disease are increasing, and they frequently require non-cardiac surgery. In Fontan physiology, a single ventricle bears the systemic and pulmonary circulation and requires higher CVP to drive the pulmonary circulation. In addition, Fontan-palliated patients are less tolerant of volume overload. Therefore, perioperative management of the post-Fontan population is challenging.

Hepatic complications related to the congestive liver caused by a high CVP are called FALD [3]. FALD consists of liver fibrosis, cirrhosis, and adenoma; focal nodular hyperplasia; and hepatocellular carcinoma [4]. FALD is associated with congestive liver and low CO [5].

The treatment of choice for FALD between laparoscopic and open abdominal surgery is often difficult to determine. In laparoscopic surgery, increased abdominal pressure caused by pneumoperitoneum can decrease venous return from the inferior vena cava. Moreover, the increased intrathoracic pressure induced by positive pressure ventilation and elevated PaCO_2_ can increase PVR. Therefore, laparoscopic surgery is considered disadvantageous in Fontan-palliated patients. Several reports have discussed laparoscopic surgery for a patient with Fontan physiology [6, 7].

However, laparoscopic surgery is less invasive than open abdominal surgery and is associated with lesser postoperative pain [2]. As inadequate pain control could increase PVR, a less-invasive approach such as laparoscopic hepatectomy is preferred. In this case, because the patient was obese (body mass index, 36.2 kg/m^2^), laparoscopic surgery had a limited effect on postoperative respiratory function, and hence, we anticipated an early recovery from surgical stress.

In this case, we chose laparoscopic surgery for FALD with strict hemodynamic management, including CO and ScvO_2_ sequential monitoring. ABP increased with CO_2_ insufflation, but CO, ScvO_2_, and CVP remained stable. After postural change to the reverse Trendelenburg position, CVP decreased. Nevertheless, CO and ScvO_2_ were not significantly affected by body position. In this Fontan-palliated patient, the postural change affected only the CVP, and the pneumoperitoneum did not influence the hemodynamic status directly. This might be due to the low intra-abdominal pressure, maintained at 8–10 cmH_2_O [7]. If circulatory dynamics become unstable because of pneumoperitoneum or postural change, we should promptly switch to open abdominal surgery.

In regard to respiratory management in this Fontan-palliated patient, we administered PEEP to prevent the exacerbation of oxygenation. Although PEEP could reduce pulmonary blood flow, hypoxia could also increase PVR and subsequently decrease pulmonary blood flow. As this patient was obese, we adopted the use of PEEP to prevent atelectasis during mechanical ventilation. Additionally, this patient inhaled NO to decrease PVR during pneumoperitoneum. In this case, the pulmonary blood flow was not monitored and so we did not correctly understand the effect of NO inhalation. However, the patient inhaled NO during pneumoperitoneum, and hemodynamic parameters, such as ScvO_2_ or CI, did not change during surgical procedures. We consider that NO could suppress the increase of PVR induced by pneumoperitoneum.

The head-up position is considered disadvantageous for Fontan-palliated patients owing to the inhibited venous return, but advantageous for laparoscopic liver resection to achieve a good surgical field. In this case, CVP decreased with postural change to the reverse Trendelenburg position. However, no changes in CO or ScvO_2_ were observed. We guess that during the reverse Trendelenburg position the intrathoracic pressure could decrease and increase pulmonary blood flow. The effect of NO is not clear, but NO could dilate the pulmonary blood vessel.

Management of low CVP during liver resection can decrease blood loss [8, 9]. The intraoperative blood loss in this case was 295 ml. The CVP reduction induced by the head-up position may have contributed to the limited bleeding. Massive blood loss could be fatal for Fontan-palliated patients, in whom adequate preload is necessary to sustain the pulmonary blood flow. If the head-up position does not affect hemodynamic parameters, it can be advantageous for laparoscopic hepatectomy in a patient with Fontan physiology. Compared to the head-down position, the intrathoracic pressure does not increase and has less impact on PVR. Increased pneumoperitoneal pressure can also control bleeding. If the pneumoperitoneal pressure exceeds the hepatic venous pressure, the oozing blood loss could decrease.

We must pay attention to the pharmacokinetics of anesthetic agents in patients with Fontan circulation. Many of Fontan-palliated people suffer from deterioration in liver function. We anesthesiologists must be careful in the administration of agents which are metabolized largely in the liver.

Consequently, laparoscopic liver resection was successfully performed in this case, with no hemodynamic complications. The patient was extubated in the operating room and shifted to the intensive care unit. Postoperative pain control was completed by epidural anesthesia. Although the patient was obese, he was discharged from the hospital on the 8th postoperative day with no respiratory complications.

## Conclusions

In the perioperative management of FALD, CO_2_ insufflation had a limited effect on hemodynamics. With strict circulation management using noninvasive monitoring systems such as the PiCCO catheter, laparoscopic surgery for a patient with Fontan physiology can be performed safely. Postural change to the reverse Trendelenburg position can decrease CVP, which could contribute to reduced blood loss. If postural change or CO_2_ insufflation affects hemodynamic status, open liver resection should be performed. Comprehensive hemodynamic assessment by noninvasive transpulmonary thermodilution in addition to myocardial motion monitoring by transesophageal echography can provide valuable information for determining the time of shift to open abdominal surgery.

## Data Availability

The datasets used and/or analyzed during the current study are available from the corresponding author upon reasonable request.

## Abbreviations

ABP: Arterial blood pressure
PaCO_2_: Partial pressure of arterial carbon dioxide
PVR: Pulmonary vascular resistance
CVP: Central venous pressure
FALD: Fontan-associated liver disease
CO: Cardiac output
ScvO_2_: Central venous saturation
SpO_2_: Saturation of peripheral oxygen
EtCO_2_: End-tidal CO_2_
CI: Cardiac index
NIBP: Noninvasive blood pressure
F_I_O_2_: Fraction of inspired oxygen
